# A turn-on fluorescent immunosensor for neurodegenerative disease related neurofilament light chain protein

**DOI:** 10.1007/s00604-025-06995-4

**Published:** 2025-02-04

**Authors:** Qingting Song, Hailong Zhang, Jia Kong, Man Shing Wong, Hung-Wing Li

**Affiliations:** 1 Department of Chemistry, The Chinese University of Hong Kong, Hong Kong, SAR China; 2 Department of Chemistry, Hong Kong Baptist University, Hong Kong, SAR China

**Keywords:** Neurodegenerative diseases, Early screening, Neurofilament light chain (NfL), Cost-effective diagnostic tool, Immunoassay, Magnetic nanoparticles

## Abstract

Neurodegenerative diseases (NDs) pose significant challenges to both longevity and quality of life, affecting millions worldwide and ranking as a major cause of global morbidity and disability. There is an urgent need for less invasive, cost-effective diagnostic methods that can reliably detect ND-related biomarkers in readily available biofluids such as serum. In response to this need, we have developed an ultra-sensitive assay that utilizes magnetic nanoparticles and a novel, tailor-made turn-on fluorescent probe named F-SPG. This innovative assay enables the detection of neurofilament light chain (NfL) at femtomolar concentrations, with a remarkable detection limit of 24 fM which is at least 18 times more sensitive than the conventional ELISA kits. Our assay’s design eliminates the need for traditional secondary antibody in immunoassay, thereby streamlining the diagnostic process. Recoveries exceeding 95% underscore the assay’s precision and reliability. It has proven effective in distinguishing Alzheimer’s Disease patients from healthy individuals by quantifying serum NfL levels, showcasing its potential as a cost-effective, ultra-sensitive tool for the early screening of neurodegenerative diseases.

**Graphical abstract:**

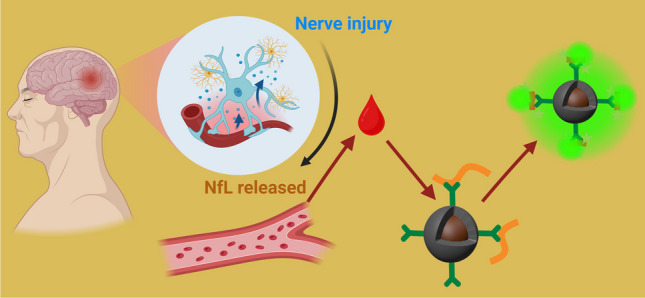

**Supplementary Information:**

The online version contains supplementary material available at 10.1007/s00604-025-06995-4.

## Introduction

As life expectancy has seen a significant rise due to scientific and medical advancements, the focus on healthcare has increasingly shifted towards improving the quality of life rather than merely extending the duration of life. This paradigm shift has steered medical practice from a traditionally reactive, treatment-based approach towards a more proactive, preventative strategy. Among the primary adversaries in the realm of chronic diseases, referred to as the “Four Horsemen of Chronic Disease” by Peter Attia, neurodegenerative diseases (NDs) pose a particularly formidable challenge to both longevity and quality of life [1]. These diseases, which include a spectrum of neurodegenerative, neuropsychiatric, and neurotraumatic disorders, represent a complex and multifaceted threat to human health. Prominent among these are Alzheimer’s disease (AD) and Parkinson’s disease (PD), which are the most prevalent forms of neurodegenerative disorders. Alone in the United States, AD and PD currently affect approximately 6.9 million and nearly 1 million individuals, respectively [2, 3]. According to a comprehensive review in *The Lancet Neurology*, neurological conditions now stand as the leading cause of morbidity and disability worldwide, impacting 43% of the global population. This prevalence places immense social and economic burdens on caregivers, healthcare systems, and communities at large. Despite the advances in medical science, the treatments available today for these conditions primarily offer temporary symptomatic relief and fail to address the underlying causes or halt disease progression. This stark reality underscores the urgent need for early diagnosis and proactive therapeutic strategies that can delay the onset and progression of these debilitating diseases, thereby not only extending lifespan but also enhancing health span, ensuring that life years gained are marked by vitality and reduced dependency.

Currently, the diagnosis of most neurodegenerative diseases (NDs) relies heavily on clinical examinations that are supported by imaging techniques such as Positron Emission Tomography (PET), Magnetic Resonance Imaging (MRI), and computed tomography (CT). These imaging modalities are invaluable for identifying abnormal brain regions indicative of neurodegeneration; however, they come with limitations including relative insensitivity, operational complexity, and high costs. Moreover, a significant challenge in diagnosing NDs is that noticeable clinical symptoms often only manifest after substantial brain damage has already occurred, making early detection, before irreversible damage sets in, particularly difficult [4, 5]. Another critical diagnostic approach involves the analysis of cerebrospinal fluid (CSF), which is a clear fluid surrounding the brain and spinal cord that provides mechanical protection and chemical stability. Because of its direct contact with brain tissues, CSF is an ideal medium for detecting pathological changes. Key biomarkers for NDs such as amyloid-beta (Aβ), phosphorylated tau (p-tau), glial fibrillary acidic protein (GFAP), and neurofilament light chain (NfL) proteins in CSF often show abnormal levels up to 15 − 30 years before the onset of clinical symptoms [6, 7]. Despite its effectiveness, the CSF test is invasive, requiring a lumbar puncture to collect the sample, and it is also associated with high costs [8]. For these reasons, there is an urgent need for the development of ultra-sensitive, specific, and cost-effective assays capable of directly detecting NDs biomarkers in easily accessible body fluids. Such advancements could revolutionize the field of neurodegenerative disease diagnostics, facilitating earlier and more accurate detection, which is crucial for timely therapeutic intervention and better management of these conditions [9]. Nonetheless, detecting subtle changes in these conveniently accessed biofluids is more challenging due to the significantly lower concentrations of disease biomarkers (serum levels are approximately 50 − 100 times lower than CSF levels) [10]. Existing techniques struggle to detect these low levels unless laborious sample pre-concentration procedures are employed, and adequately sensitive methods are still lacking. Therefore, an ultra-sensitive, specific, and cost-effective assay for the direct detection of NDs biomarkers in body fluids is urgently needed and holds great promise.

Neurofilament light chain (NfL, ~ 68 kDa) is the smallest component of the neurofilament triplet proteins, which are crucial structural elements of axons in the central nervous system (CNS) [11]. When neuronal tissues in the CNS are damaged due to aging, trauma, or disease, neurofilaments are released into the CSF and, to a lesser extent, into the bloodstream. Elevated neurofilament levels have been observed in the CSF and blood of patients with neurological conditions such as AD, PD, and HD compared to age-matched controls [12, 13]. The detection of NfL has emerged as a vital biomarker for neurodegeneration due to its strong correlation with neuronal damage and axonal degeneration across various neurological disorders. Studies have demonstrated that increased levels of NfL in both CSF and plasma are indicative of disease severity and progression, making it a valuable tool for early diagnosis and monitoring therapeutic responses. Furthermore, NfL detection provides insights into the underlying neurodegenerative processes, allowing for better differentiation between various neurological conditions. Although NfL is non-specific and only indirectly related to the pathogenesis of these diseases, its ability to reflect active neuronal damage underscores its importance in clinical settings. As such, NfL has become recognized not only as a potential biomarker for neurodegeneration but also as a critical component in advancing our understanding of neurological diseases and improving patient outcomes [14–18].

To measure NfL levels, conventional methods like the enzyme-linked immunosorbent assay (ELISA) [19, 20] and electro-chemiluminescence (ECL) assay [12, 21] are widely used, particularly in CSF, where they can detect NfL concentrations ranging from dozens to hundreds of pg/ml. However, these methods face significant limitations in sensitivity when applied to blood samples, with more than 50% of plasma samples failing to be reliably quantified [22]. This limitation highlights the necessity for more sensitive techniques in clinical settings for accurate plasma NfL quantification. The single molecule array (SiMoA) is the most developed ultra-sensitive technology for blood-derived biomarkers. In the literature, the SiMoA technology implemented on the Quanterix® (Billerica, MA, USA) Simoa™ device is the most frequently cited method for NfL quantification in serum and CSF [23–25]. This method has been shown to offer significantly higher sensitivity for detecting blood-derived biomarkers, with studies demonstrating that SiMoA is 126 times more sensitive than ELISA and 25 times more sensitive than ECL assays. This superior sensitivity makes it a preferred choice for quantifying NfL levels in both serum and CSF [22]. More recently, an immunoassay based on a microfluidic approach, deployable on the Ella™ device from Protein Simple®, have become available for quantifying plasma NfL, albeit with higher measured levels and greater variability compared to SiMoA technology [26]. However, both SiMoA and the microfluidic-based assays require specialized equipment exclusive to their respective manufacturers, which can limit their accessibility in some clinical environments. Thus, the development and adoption of these ultra-sensitive methodologies are critical for improving the detection and monitoring of neurodegenerative diseases in clinical practice.

**Scheme 1:**
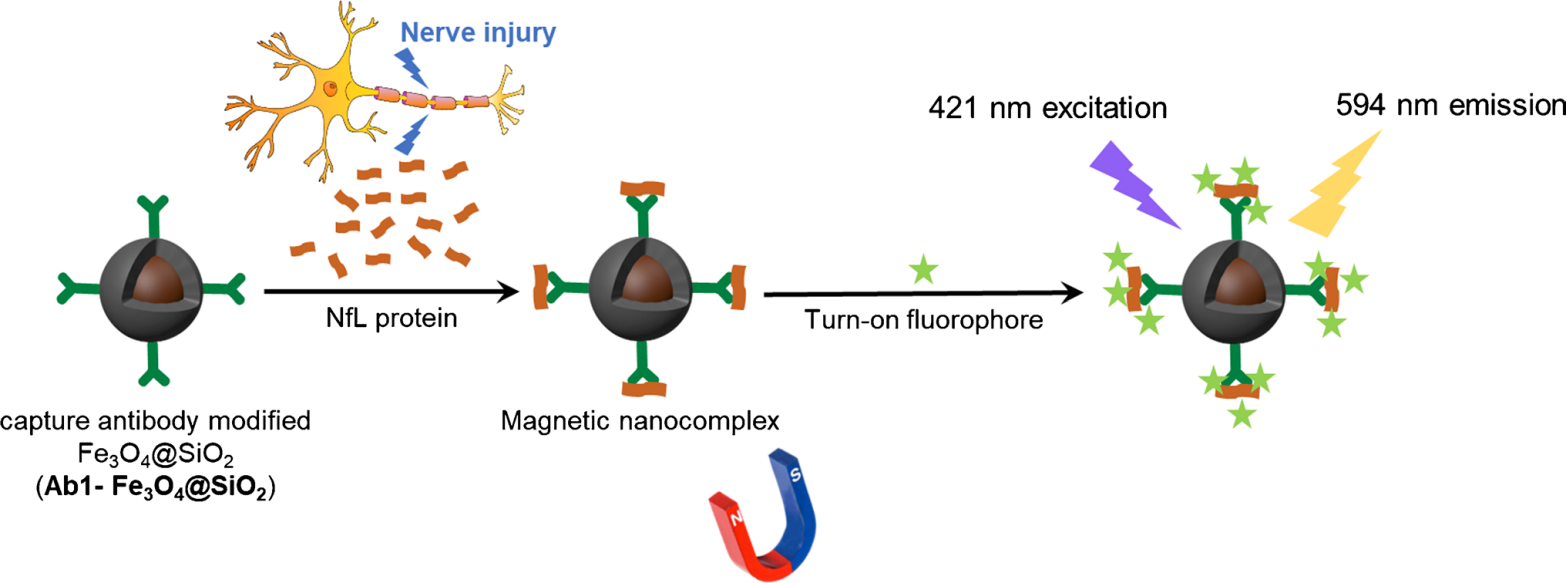
Schematic illustration of employing capture antibody-modified magnetic nanoparticles (Ab1-Fe_3_O_4_@SiO_2_) to detect NfL

Herein, we develop an ultra-sensitive serum NfL detection assay for diagnosing neurodegenerative diseases (NDs), utilizing a novel magnetic nano platform. As illustrated in Scheme 1, we begin by employing magnetic nanoparticles that have been modified with capture antibodies, termed nanoprobes, to selectively bind NfL proteins and form immunocomplexes. Thanks to the super-paramagnetic properties of these nanoparticles, the captured analyte molecules can be easily manipulated. For instance, rinsing and preconcentration can be readily achieved by placing a magnet near the reaction micro-vial, thereby preventing any loss of sample. Following capture, these immunocomplexes are labeled with a specially designed fluorophore, *F-SPG*, which exhibits a significant increase in fluorescence upon binding to the protein-based immunocomplex, thereby facilitating signal readout. This fluorescence quantitatively indicates the presence of trace target proteins. In this assay, the magnetic nanoparticles perform a dual role by capturing and preconcentrating target proteins on their surfaces, which facilitates the separation of magnetic immunocomplexes from undesired reactants through the application of a magnet. The fluorophore F-SPG is specifically engineered to enhance fluorescence upon binding to the target proteins, resulting in a significant increase in signal visibility. The integration of magnetic separation for targeted capture and fluorescence enhancement for signal amplification, combined with the inherent specificity of the immunoassay, ensures high sensitivity of our method, achieving detection limits as low as 24 fM. When compared to traditional commercial ELISA kits, our assay requires significantly smaller sample volumes and simplifies the detection process by eliminating the need for a detection antibody. Quantitative analysis of serum NfL levels using our assay successfully distinguishes between Alzheimer’s Disease patients and healthy individuals, highlighting the assay’s potential as a cost-effective, ultra-sensitive tool for clinical ND screening. This platform's efficacy suggests it could be a valuable addition to current diagnostic methodologies, enhancing early detection and treatment strategies for neurodegenerative diseases.

## Materials and methods

### Chemicals and instruments

Tetraethyl orthosilicate (TEOS, 99%), (3-Aminopropyl)triethoxysilane(APTES, 99%), and glutaraldehyde (GA, Grade I) were purchased from Sigma (St. Louis, USA). Anti-NfL capture antibody (Ab1, ab280029), anti-NfL detection antibody (Ab2, ab280028), and NfL (ab224840) were purchased from Abcam (Cambridge, UK). The human serum is purchased from PrecisionMed (US). Fe3O4 nanoparticles were purchased from RuixiBiotech (Xi’an, China). All other chemicals were of analytical purity. The fluorescence intensity was measured by a FS5 Spectrofluorometer from Edinburgh Instrument (Livingstone, UK).

### Molecular docking simulation

Molecular docking simulations were conducted utilizing Autodock Vina version 1.2.0 [27]. The structural model of the NfL, predicted by AlphaFold (https://alphafold.ebi.ac.uk/entry/P07196) was employed alongside the F-SPG as the initial conformation for docking. Prior to docking, both the protein and ligand structures underwent a preparation process using AutoDock Tools. The entire protein structure was designated as the docking site. From the docking simulations, the model exhibiting the highest binding affinity was selected to represent the protein–ligand complex.

### Synthesis and characterization of silica-coated iron oxide nanoparticles (Fe_3_O_4_@SiO_2_ NPs)

The sol–gel reaction was used to coat the silica. Add a mechanically stirred mixture of 5 mL of 28% NH_4_OH solution, 29 mL of distilled water, 27.5 mL of ethanol, and 1 mL of Fe_3_O_4_ NPs previously diluted with 9 mL ethanol of the 1 mL stock solution. Then, 1.5 mL TEOS was diluted with 28.5 mL ethanol solution and added to the mechanically agitated solution drop by drop. Then, the solution was stirred continuously for up to four hours. Finally, the NPs were washed three times with distilled water and three times with ethanol before being redistributed in 7.5 mL of ethanol. The morphology of the Fe_3_O_4_@SiO_2_ NPs was determined by high-resolution transmission electron microscopy (TEM) (JEOL JEM-ARM200F, Japan). Solution properties such as hydrodynamic diameters and zeta potentials were evaluated using a Malvern Zetasizer instrument (Nano ZS90, UK).

### Construction of nanoprobes

The capture antibody was coupled with the silica-coating Fe_3_O_4_ NPs through the crosslinker glutaraldehyde. 5 mg Fe_3_O_4_@SiO_2_ NPs were added to 1 mL APTES and 2 ml ethanol solution and rotated at 75 °C for 24 h. The obtained NPs were cleaned twice with ethanol and water successively and redispersed in DI water. The NPs were then functionalized with 250 μL GA and vortexed at room temperature for 2 h. NPs were sequentially washed twice with water and PBS and redispersed in PBS. Finally, the prepared nanoparticles were incubated with capture antibodies (Ab1) for 1.5 h. The resulting magnetic nanoprobes were separated from the solution using a magnet and redispersed in 500 μL PBS.

### Optimization of the magnetic immunoassay conditions

The series of optimization experiments were methodically executed by altering a solitary variable at a time, with performance evaluated by comparing the fluorescence intensity of the fluorophore at its maximum emission wavelength of 594 nm. Specifically, to evaluate the optimal coverage of Ab1 on the NPs, concentrations of 1, 3, and 5 nM of Ab1 were introduced to conjugate with the NPs, thereby synthesizing nanoprobes with different Ab1 density on the surface. Subsequently, to fine-tune the nanoprobes’ concentration conducive for the detection assay, disparate concentrations, namely 1, 2, and 4 mg/ml of nanoprobes, were employed in a co-incubation with the targets. Moreover, to determine the precise incubation period requisite for optimal capture efficiency, the nanoprobes were subjected to incubation at 25 °C with the analytes for durations of 30, 60, and 90 min. Furthermore, in pursuit of identifying the ideal concentration of the turn-on fluorophore essential for efficacious labeling, the immunocomplexes were incubated with varying concentrations of 10, 30, and 50 μM of the fluorophore. Lastly, to refine the detection methodology, a comparative analysis was conducted by co-incubating the nanoprobes with the targets, both in the absence and presence of 600 pM detection antibodies (Ab2), thereby elucidating the distinction in detection efficiency.

### Detection of the target protein biomarker

Each condition selected for detection represented the pinnacle of optimization. The external calibration curve was formulated by mapping the fluorescence intensities obtained at the peak emission wavelengths (594 nm) against various analyte concentrations. Specifically, predetermined NfL concentrations were co-incubated with 1 mg/ml nanoprobes—prepared by conjugating Fe_3_O_4_@SiO_2_ nanoparticles with 3 nM Ab1—for 60 min to form immunocomplexes. These resultant magnetic immunocomplexes were then isolated by discarding the supernatant through magnetic separation, followed by dual washes in PBS. Subsequently, a 30 μM solution of the turn-on fluorophore, F-SPG, was introduced to label the complexes. After a brief 10-min incubation period, fluorescence measurement was undertaken using an Edinburgh Spectrofluorometer instrument (FS5 Spectrofluorometer, UK). The fluorescent signal at 594 nm was recorded for quantification.

### Selectivity of the nanoprobes

To evaluate the selectivity of the detection assay, nanoprobes were incubated with 500 fM NfL, 5 pM Aβ40 monomer, 5 pM Aβ42 monomer, 5 pM tau and 2 pM phosphorylated tau (p-tau), respectively, under optimal conditions. The resultant complexes were then labeled with a 30 μM solution of F-SPG. The selectivity was assessed by comparing the fluorescence intensities of F-SPG at the peak emission wavelength of 594 nm.

### Quantification of NfL in human serum

Human serum was initially directly diluted by tenfold with PBS (10mM, pH 7.4). After establishing the external calibration curve by correlating the average fluorescence intensity with different final concentrations of NfL proteins, the average fluorescence intensity of serum sample with unknown concentration is compared with the standard curve to determine the content of the target protein in the sample.

## Results and discussion

### Design and synthesis

**Fig. 1:**
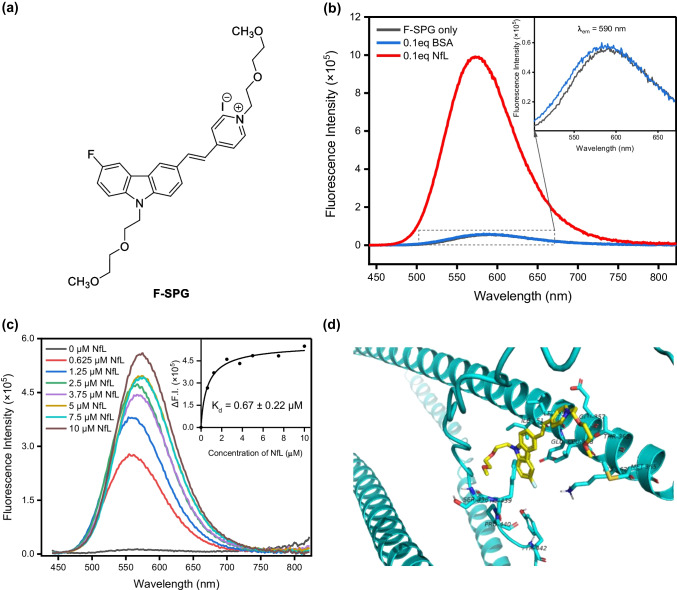
**a** Chemical structure of newly developed fluorophore, F-SPG. **b** Fluorescence spectra of F-SPG (30 μM) in the absence and presence of NfL and BSA (3 μM) in pH7.4 phosphate buffer when excited at 421 nm. **c** Fluorescence titration spectra of F-SPG (1µM) upon an addition of various concentrations of NfL in 25 mM phosphate buffer (pH = 7.4). The insert shows the curve fitting of the nonlinear analysis of intensity difference. **d** Molecular docking simulation done by the Autodock Vina program (version 1.2.0) showing the interaction of F-SPG with the binding site of NfL residue which is predicted by AlphaFold (https://alphafold.ebi.ac.uk/entry/P07196)

The fluorophore F-SPG, a target-activatable turn-on probe, plays a crucial role in signal transduction for this assay. Developed from a versatile carbazole-derived cyanine structure, F-SPG exhibits a strong fluorescence enhancement upon binding to the neurofilament light chain (NfL). The synthesis and structure characterization of F-SPG are summarized in the Supporting Information (Scheme S1 and Figure S1). It absorbs prominently at 421 nm (Figure S2) and initially emits weak fluorescence at 590 nm in a pH 7.4 phosphate buffer. However, the addition of 0.1 equivalent of NfL triggers a remarkable 20-fold increase in fluorescence, while similar enhancements are not observed with bovine serum albumin (BSA), highlighting the specific turn-on capability of NfL (Fig. 1b). This substantial fluorescence increase is attributed to the strong interaction between F-SPG and NfL, which restricts the rotational freedom of the F-SPG excimer, thus reducing radiationless decay and enhancing fluorescence. F-SPG's binding affinity to NfL was quantified through fluorescence titration, resulting in a dissociation constant (K_d_) of 0.67 µM, as determined by Scatchard plot analysis (Fig. 1c). This indicates a strong interaction between the fluorophore and NfL. The predicted interactions between F-SPG (highlighted in yellow) and NfL (depicted in cyan) have been substantiated through molecular docking techniques utilizing Autodock Vina program (Fig. 1d). The determined docking score of −10.642 kcal/mol supports the binding potential, which is attributed to the formation of hydrogen bonds, van der Waals forces, and various interactions such as pi-Anion, pi-Amide, and pi-Alkyl. These interactions involve a series of residues, namely Glu355, Ile351, Leu354, Glu357, Leu358, Thr361, Lys362, Met365, Pro440, Ser438, Phe439, and Tyr442, confirming the robust molecular engagement between F-SPG and NfL. These results support the use of F-SPG to label immunocomplexes effectively and amplify fluorescent signal significantly in our detection assay.

**Fig. 2:**
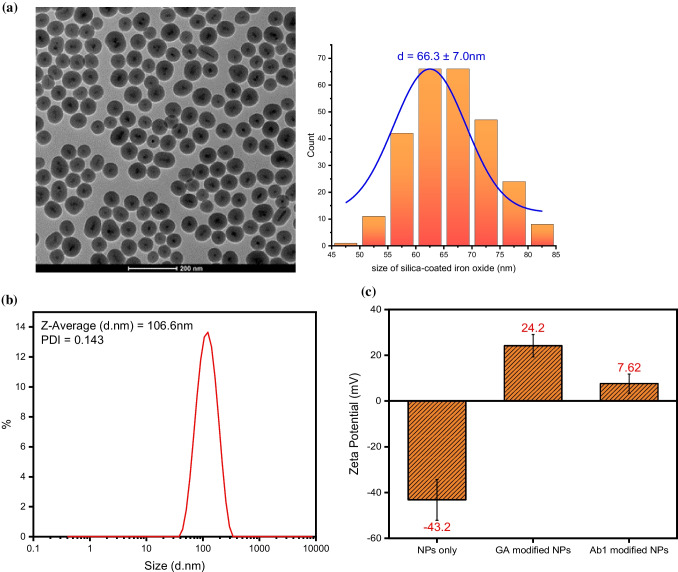
**a** Transmission electron microscopy (TEM) image of silica-coating Fe_3_O_4_ NPs (left) and size distribution of silica-coated iron-oxide nanoparticles (right). **b** Hydrodynamic size of Fe_3_O_4_@SiO_2_ NPs. **c** Zeta potential of the bare Fe_3_O_4_@SiO_2_ NPs (NPs only), APTES and GA modified Fe_3_O_4_@SiO_2_ NPs (GA modified NPs), and capture antibody conjugated Fe_3_O_4_@SiO_2_ NPs (Ab1 modified NPs)

Regarding the detection strategy, the assay begins with the synthesis of silica-coated iron oxide nanoparticles (Fe_3_O_4_@SiO_2_ NPs). These nanoparticles were then functionalized by conjugating with APTES-activated Fe_3_O_4_@SiO_2_ NPs with a monoclonal capture antibody against NfL, using glutaraldehyde as a crosslinker (Figure S3). The spherical morphology of Fe_3_O_4_@SiO_2_ NPs and their size distribution, with an average diameter of approximately 66.3 ± 7.0 nm, were confirmed by TEM imaging (Fig. 2a). Dynamic light scattering (DLS) tests showed that the nanoparticles have a hydrodynamic size of ~ 106 nm in aqueous solution with a polydispersity index of 0.143, indicating uniform dispersion (Fig. 2b). The successful preparation of the nanoprobes was evident from changes in the surface charge, monitored throughout the process. Initially highly negative, the zeta potential of bare Fe_3_O_4_@SiO_2_ NPs shifted to a relatively positive value after modification with APTES and glutaraldehyde due to the presence of aldehyde groups. Following the immobilization of the capture antibody, the zeta potential decreased from 24.2 to 7.62 mV, indicating successful antibody attachment (Fig. 2c). These nanoprobes exhibit robust magnetic properties, allowing for efficient sample manipulation using a magnet bar, as demonstrated by their rapid attraction to the magnet side within 30 s (Figure S4). The magnetic nanoprobes are subsequently incubated with NfL proteins and detection antibodies sequentially to form magnetic immunocomplexes. These complexes are then labeled by the fluorophore, F-SPG, after thorough rinsing and preconcentrating, setting the stage for the final fluorescence-based detection.

### Optimization of the immunoassay

The exceptional sensitivity of the detection assay is crucial for accurately quantifying protein biomarkers that are present only in trace amounts in the body fluids of individuals with neurodegenerative diseases (NDs), particularly in the early stages of the disease. To enhance both detection efficiency and assay sensitivity, a series of optimization experiments were conducted. The increase in fluorescence signal (ΔF.I.) in the presence of the target, compared to conditions without the target (ΔConcentration), served as an indicator of the performance of the detection method.

**Fig. 3:**
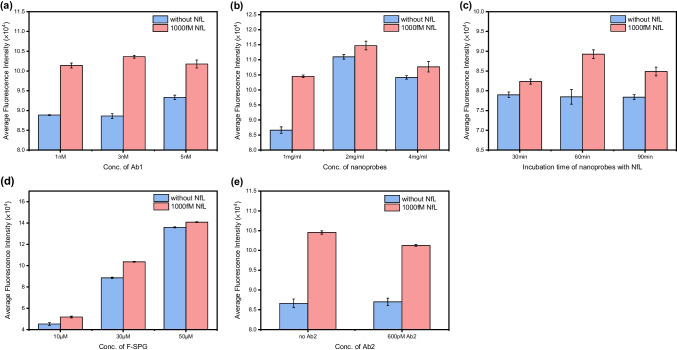
Optimization of **a** concentration of capture antibody (Ab1), **b** concentration of capture antibody-conjugated nanoprobes, **c** incubation time of nanoprobes with NfL, **d** concentration of fluorophore (F-SPG) and **e** concentration of detection antibody (Ab2). Error bars, standard error of the mean; *n* = 3

Initially, to optimize the coverage of capture antibodies (Ab1) on the nanoparticles (NPs) for maximal target capture efficiency, the nanoprobes were synthesized by incubating NPs with Ab1 at concentrations of 1 nM, 3 nM, and 5 nM, followed by incubation with an equivalent concentration of neurofilament light chain (NfL). It is essential to find a balance between antibody coverage and the capturing efficiency of the nanoprobes. As illustrated in Fig. 3a, an Ab1 concentration of 3 nM is sufficient for synthesizing effective nanoprobes, as confirmed by highest signal increase observed. Higher concentrations of Ab1 could lead to antibody overload on the NPs’ surface, potentially reducing target capture efficiency by interfering with the antibody-antigen recognition process. Furthermore, excessive concentrations of Ab1 may cause increased background fluorescence in the absence of targets, as the fluorophore’s fluorescence might be nonspecifically activated by Ab1.

An optimal nanoprobe concentration is critical not only to prevent the aggregation of magnetic immunocomplexes but also to ensure sufficient reaction platforms are available for capturing protein targets. Figure 3b shows that a nanoprobe concentration of 1 mg/ml is optimal, resulting in the most significant fluorescence enhancement. Additionally, the incubation time between the nanoprobes and the target significantly affects the performance of the detection assay. Insufficient incubation time may result in incomplete capture of free targets by the nanoprobes, while overly prolonged incubation could impair the activity of Ab1. As depicted in Fig. 3c, an incubation period of 60 min with the NfL proteins yields the highest fluorescence enhancement and was therefore selected for the detection assay.

Generally, the fluorescence signal increases with the concentration of the fluorophore. The immunocomplexes were incubated with 10, 30, and 50 μM of the protein turn-on fluorophore. As shown in Fig. 3d, the fluorescence intensity rose as the fluorophore concentration increased from 10 to 50 μM. However, higher fluorophore levels contribute to increased background noise, potentially affecting the assay’s sensitivity. Thus, a fluorophore concentration of 30 μM was chosen to label the formed immunocomplexes. Given the common use of a secondary antibody (Ab2) in other immunoassays, we investigated whether Ab2 could enhance the performance of our assay. Comparing the group without Ab2 to the group containing 600 pM Ab2, the results indicated that the absence of Ab2 led to a higher fluorescence enhancement (Fig. 3e). This may be due to the inefficacy of Ab2 in turning-on the fluorophore, while also consuming the fluorophore and blocking the binding of NfL to the fluorophore. Consequently, the addition of Ab2 was omitted from our assay to streamline the detection process.

### Performance of the immunoassay

**Fig. 4:**
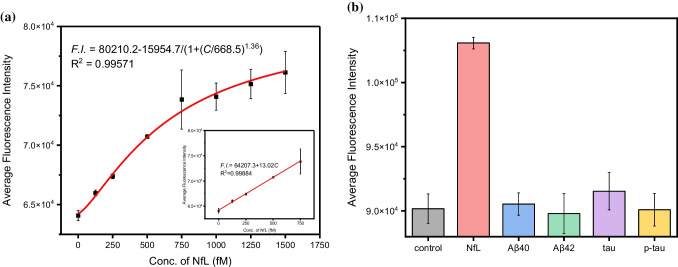
**a** Dependence of fluorescence intensity on concentration of NfL. The nanoprobes were incubated with NfL at concentrations of 0, 125, 250, 500, 750, 1000, 1250, and 1500 fM, respectively, under optimal conditions. The inset shows the corresponding linear relationship in the range of 0–750 fM. Error bars, standard error of the mean; *n* = 3. **b** Selectivity of the nanoprobes for 500 fM NfL against 5 pM Aβ40 monomer, 5 pM Aβ42 monomer, 5 pM tau and 2 pM p-tau

To validate the performance of the developed assay, we assessed its sensitivity, selectivity, and accuracy. Figure 4a illustrates the correlation between fluorescence intensity (F.I.) and the concentration of neurofilament light chain (NfL), revealing a linear relationship within the range of 0–750 fM, with a robust coefficient of determination (*R*2 = 0.99). The computed relative standard deviation (RSD) ranged from 0.61 to 3.38%, indicating that the immunosensor exhibits strong repeatability (Figure S5). Impressively, the detection limit (LOD) of this assay is as low as 24 fM (LOD = 3.3σ/S where S is the slope of the calibration curve and σ is the standard deviation of the response), which is nearly 20 times more sensitive than the commercial Human NfL ELISA Kit (ab288171 from Abcam, utilizing the same antibodies as ours), thus meeting the stringent requirements for practical applications. The specificity of the nanoprobes is crucial for the assay’s precision, especially given the variety of protein types present in human serum. To confirm the specificity of our assay, we tested several other common Alzheimer’s disease protein biomarkers (beta amyloid Aβ_40_, Aβ_42_, tau, and phosphorylated tau) that coexist with NfL in human serum as potential interferents. As shown in Fig. 4b, the fluorescence intensity fluctuations remained minimal even at significantly higher concentrations of these interfering proteins, demonstrating the assay’s exceptional selectivity. The accuracy of the method was evaluated using the standard addition technique. As detailed in Table 1, the recoveries of NfL in diluted human serum samples exceeded 95%, confirming the assay’s reliability and its potential for real sample analysis.

**Table 1:** Recoveries of NfL in diluted human serum samples

Spiked/fM	Measured/fM	Recovery/%	RSD
0	106 ± 15	——	13.8%
50	154 ± 5	96	3.3%
100	216 ± 20	110	9.3%

### Quantification of NfL in human serum samples

**Fig. 5:**
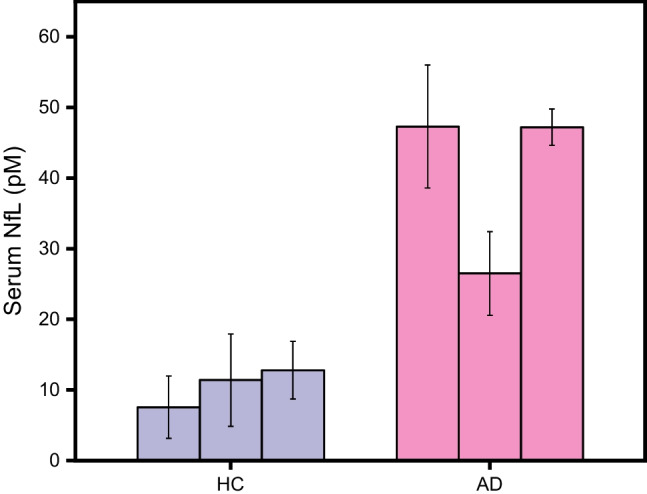
Quantification of NfL in serum samples from healthy controls and AD patients

Given that Alzheimer’s Disease (AD) is the most prevalent form of neurodegenerative diseases (NDs), it was chosen as the model disease for this study. We used the assay to measure serum neurofilament light chain (NfL) levels in three neurologically healthy individuals (HC #8346, #8353, and #8357) and three patients diagnosed with AD (AD #8749, #8696, and #8788), employing the calibration curve method. The NfL concentrations for each sample are detailed in Table 2. Notably, the RSD values for the AD patient group were much lower than those for the healthy control group, indicating improved repeatability of the assay at higher analyte concentrations. Figure 5, which is based on these results, shows that the serum NfL concentrations in healthy controls are approximately four times lower than those in individuals with AD. This significant difference confirms our assay’s ability to distinguish between individuals with AD and those without neurological impairments by measuring serum NfL levels. This finding underscores the assay’s potential utility for neurodegenerative disease screening in a clinical setting.

**Table 2:** Concentration of NfL in serum samples from AD patients and healthy controls

Sample		Measured (fM)	Calculated in serum (pM)	Average (pM)
AD	#8749	189 ± 35	47 ± 9	40 ± 10
	#8696	106 ± 24	27 ± 6	
	#8788	189 ± 10	47 ± 3	
HC	#8346	30 ± 18	8 ± 4	11 ± 2
	#8353	46 ± 26	11 ± 7	
	#8357	51 ± 16	13 ± 4

## Conclusions

In this study, we developed an ultra-sensitive diagnostic assay for NDs using a magnetic nano-platform. Our assay utilizes magnetic nanoparticles modified with capture antibodies, referred to as nanoprobes, to isolate and detect NfL proteins. The superparamagnetic properties of these nanoparticles enable efficient handling and concentration of the sample with minimal loss, using a magnet. The immunocomplexes are then labeled with a specially designed fluorophore that displays significant fluorescence enhancement upon binding. This dual mechanism enhances the assay's sensitivity and specificity, achieving detection limits as low as 24 fM, which significantly outperforms commercial ELISA kits (as listed in Table 3). Notably, our assay requires only minimal sample volumes and simplifies the overall protocol by eliminating the need for detection antibodies. Verification was conducted by determining NfL levels in spiked serum samples, yielding recoveries exceeding 95%. Its effectiveness was demonstrated by differentiating between AD patients and healthy controls through the quantification of serum NfL levels. These results highlight the assay’s potential as a cost-effective and ultra-sensitive tool for the clinical screening of NDs. Additionally, the flexibility of our platform allows for the investigation and quantification of other protein biomarkers by simply switching the specific antibodies and fluorophores used. Given that a combination of biomarker profiles significantly enhances the reliability and accuracy of NDs diagnosis, future research should aim to expand this immunoassay-based method to detect a broader array of ND biomarkers.

**Table 3:** Limits of detection (Lods) of various assay platforms

Assay platform	LoD	Ref
ELISA	1.24 pM	[28]
ELISA	3.65 pM	[29]
UmanDiagnostics NF-light assay ELISA	1.03 pM	[22]
Abcam Human NEFL ELISA Kit	450 fM	[30]
Simoa NfL assay	9.06 fM	[22]
ECL	228 fM	[12]
Magnetic NPs-based Fluorescent Immunosensor	23.7 fM	This work

## Supplementary Information

Below is the link to the electronic supplementary material.

Supplementary file1 (PDF 504 KB)

## Data Availability

No datasets were generated or analysed during the current study.
