# Supplementary material for: A turn-on fluorescent immunosensor for neurodegenerative disease related neurofilament light chain protein
Source: Mikrochim Acta. 2025 Feb 4;192(3):131. doi: 10.1007/s00604-025-06995-4 (PMC11794385; doi:10.1007/s00604-025-06995-4)
Supplement: Supplementary file 1 — Supplementary file1 (PDF 504 KB) [file 604_2025_6995_MOESM1_ESM.pdf]

Supporting information

A Turn-on Fluorescent Immunosensor for Neurodegenerative Disease Related Neurofilament Light Chain Protein

Qingting Song[1], Hailong Zhang[2], Jia Kong[2], Man Shing Wong[2, *] and Hung Wing LI[1, *]

[1]Department of Chemistry, The Chinese University of Hong Kong, Sha Tin, HONG KONG SAR, China

[2]Department of Chemistry, Hong Kong Baptist University, Kowloon Tong, Hong Kong SAR, China

[*]Corresponding author: Man Shing Wong, E-mail: mswong@hkbu.edu.hk; Hung Wing LI, E-mail: hungwingli@cuhk.edu.hk

List of Contents

Scheme S1. The synthetic route of **F-SPG**. S5

Figure S1. Characterization of **F-SPG**. ^{1}H NMR, ^{13}C NMR and HRMS Spectra of F-SPG. S7

Figure S2. Absorption and emission spectrum of F-SPG in a pH 7.4 phosphate buffer. S7

Figure S3. Preparation process of nanoprobes, Ab1-Fe_3O_4@SiO_2.. S8

Figure S4. Magnetic properties of the nanoprobes. S8

References... S9

Experimental section

2-(Methoxymethoxy)ethyl 4-methylbenzenesulfonate (1). To the solution of 2-(2-methoxyethoxy)ethan-1-ol (10.57 g, 88 mmol) in THF (100 mL) was added tosyl chloride (15.25 g, 80 mmol). After cooling to 0 °C, 80 mL of 10% NaOH solution was then added. After stirring at room temperature for 3 h, the reaction mixture was carefully quenched with water and extracted with ethyl acetate three times. The combined organic phase was washed with water and then brine. Organic layer was dried over anhydrous sodium sulfate, filtered, and concentrated in vacuo to afford **1** (17.28 g) as colorless oil in 83% yield. ^{1}H NMR (400 MHz, $CDCl_3$, δ): 7.78 (d, *J* = 8.4 Hz, 2H), 7.32 (d, *J* = 8.5 Hz, 2H), 4.15 (t, *J* = 4.9 Hz, 2H), 3.67 (t, *J* = 4.8 Hz, 2H), 3.58-3.53 (m, 2H), 3.49-3.44 (m, 2H), 3.33 (s, 3H), 2.43 (s, 3H).

1-Iodo-2-(2-methoxyethoxy)ethane (2). To the solution of **1** (2.6 g, 10 mmol) in acetone (25 mL), sodium iodide (1.8 mg, 12 mmol) was added. After stirring the reaction mixtures at room temperature under nitrogen in the dark for 24 hours. the reaction mixture was filtered. Then the solvent was removed. The residue was purified by silica gel chromatography using petroleum ether and ethyl acetate as eluent (PE : EA = 100:1) to afford **2** (2.13 g) as colorless oil in 93% yield. ^{1}H NMR (400 MHz, $CDCl_3$, δ): 3.77 (t, *J* = 6.84 Hz, 2H), 3.67 (m, 2H), 3.57 (m, 2H), 3.40 (s, 3H) 3.28 (t, *J* = 7.12 Hz, 2H).

1-(2-(2-Methoxyethoxy)ethyl)-4-methylpyridin-1-ium (3). The mixture solution of **2** (1.38 g, 6 mmol) and 4-methylpyridine (465.3 mg, 5 mmol) in acetonitrile (10 mL) in sealed tube was heated to 80 ℃ for 12 h. After cooling to room temperature, the organic solvent was removed. The crude product was purified by recrystallization from methanol and ethyl acetate to afford **3** in 89% yield. ^{1}H NMR (400 MHz, DMSO-d_6, δ): 8.79 (d, *J* = 6.8 Hz, 2H), 7.95 (d, *J* = 6.3 Hz, 2H), 5.19 (s, 3H), 4.53 (t, *J* = 5.3 Hz, 2H), 3.90 (t, *J* = 5.3 Hz, 2H), 3.53-3.49 (m, 2H), 3.41-3.37 (m, 2H), 3.27 (s, 3H).

2-Chloro-N-(4-fluorophenyl)aniline (4). To the solution of 2-chloroaniline (6.12 g, 48 mmol) in toluene (100 mL) was added 1-bromo-4-fluorobenzene (7 g, 40 mmol), sodium tert-butoxide (7.69 g, 80 mmol), binaphthyl-2.2'-diphemyl phosphine (2.49 g, 4 mmol) and $Pd(OAc)_2$ (449 mg, 2 mmol). The resulting mixture was heated to reflux overnight under a nitrogen atmosphere. After cooling to room temperature, the reaction mixture was carefully quenched with water, filtered and extracted with ethyl acetate three times. The combined organic phase was washed with water and then brine. Organic layer was dried over anhydrous sodium sulfate and the solvent was removed. The residue was purified by silica gel chromatography using petroleum ether and ethyl acetate as eluent (PE : EA = 50:1) to afford **4** (7.27 g) as colorless oil in 82% yield. ^{1}H NMR (400 MHz, $CDCl_3$, δ): 7.37-7.32 (m, 1H), 7.19-7.00 (m, 6H), 6.83-6.75 (m, 1H), 6.02 (s, 1H).

3-Fluoro-9H-carbazole (5). 4 (6.63 g, 30 mmol), K_2CO_3 (12.44 g, 90 mmol), binaphthyl-2.2'-diphemyl phosphine (1.87 g, 3 mmol), $Pd(OAc)_2$ (337 mg, 1.5 mmol). were heated to 155 ℃ in 150 mL of DMF for overnight with stirring under a nitrogen atmosphere. After cooling to room temperature, the reaction mixture was carefully quenched with water, filtered and extracted with ethyl acetate three times. The combined organic phase was washed with water and then brine. Organic layer was dried over anhydrous sodium sulfate and the solvent was removed. The residue was purified by silica gel chromatography using petroleum ether and ethyl acetate as eluent (PE : EA = 30:1) to afford **5** (3.94 g) as a white solid in 71% yield. ^{1}H NMR (400 MHz, $CDCl_3$, δ): 8.06-7.99 (m, 2H), 7.73 (dd, *J* = 8.9,2.5 Hz, 1H), 7.47-7.41 (m, 2H), 7.34 (dd, *J* = 8.7,4.2 Hz, 1H), 7.25-7.21 (m, 1H), 7.16 (dt, *J* = 9.0,2.5 Hz, 1H).

3-Bromo-6-fluoro-9H-carbazole (6). 5 (3.7 g, 20 mmol) was dissolved in THF (100 mL). After cooling to 0 °C, N-bromosuccinimide (3.56 g, 20 mmol) in 60 ml THF was carefully added dropwise into the solution over 1 h. After stirring for 1 h at 0 °C, the reaction mixture was carefully quenched with water and extracted with ethyl acetate three times. The combined organic phase was washed with water and then brine. Organic layer was dried over anhydrous sodium sulfate and the solvent was removed. The residue was purified by silica gel chromatography using petroleum ether and ethyl acetate as eluent (PE : EA = 20:1) to afford **6** (5.18 g) as a white solid in 98% yield. ^{1}H NMR (400 MHz, $CDCl_3$, δ): 8.13 (d, *J* = 1.9 Hz, 1H), 8.06 (s, 1H), 7.66 (dd, *J* = 8.8, 2.5 Hz, 1H), 7.51 (dd, *J* = 8.5, 1.9 Hz, 1H), 7.35 (dd, *J* = 8.8, 4.2 Hz, 1H), 7.31 (d, *J* = 8.7 Hz, 1H), 7.18 (dd, *J* = 9.0, 2.5 Hz, 1H).

3-Bromo-6-fluoro-9-(2-(2-methoxyethoxy)ethyl)-9H-carbazole (7). A solution mixture of compound 6 (6.69 g, 18

mmol), 1 (7.03 g, 24 mmol) and KOH (5.6 g, 100 mmol) in THF (100 mL) was heated to reflux for 3 h. After cooling down to room temperature, the reaction mixture was carefully quenched with water and extracted with dichloromethane three times. The combined organic phase was washed with water and then brine. Organic layer was dried over anhydrous sodium sulfate and the solvent was removed. The residue was purified by silica gel chromatography using petroleum ether and dichloromethane as eluent (PE : DCM = 1:1) to afford 7 (5.73 g) as a white solid in 87% yield. 1H NMR (400 MHz, CDCl3, δ): 8.12 (d, J = 1.5 Hz, 1H), 7.67 (dd, J = 8.8, 2.2 Hz, 1H), 7.54 (dd, J = 8.6, 1.9 Hz, 1H), 7.39 (dd, J = 8.9, 4.1 Hz, 1H), 7.34 (d, J = 8.4 Hz, 1H), 7.21 (dt, J = 9.0, 2.5 Hz, 1H), 4.46 (t, J = 5.9 Hz, 2H), 3.84 (t, *J* = 5.9 Hz, 2H), 3.51-3.47 (m, 2H), 3.41-3.37 (m, 2H), 3.29 (s, 3H).

6-Fluoro-9-(2-(2-methoxyethoxy)ethyl)-9H-carbazole-3-carbaldehyde (8). To a solution of **7** (5.05 g, 16 mmol) in dried THF (80 mL) was added *n*-BuLi (12.9 mL 19.2 mmol) at -78 °C. The resulting mixture was stirred at -78 °C for 1 h and then added with N-formylmorpholine (3.2 mL, 32 mmol). The reaction mixture was allowed warming up to room temperature and stirred overnight before quenching with aqueous ammonia chloride solution. Water was added and extracted with ethyl acetate three times. The combined organic phase was washed with brine and dried over anhydrous sodium sulfate. After removing the solvent, the residue was purified by silica gel chromatography using ethyl acetate and petroleum ether (EA: PE = 1: 3) as eluent to afford **8** (2.83 g) as a white solid in 56% yield. ^{1}H NMR (400 MHz, CDCl3, δ): 10.09 (s, 1H), 8.54 (d, *J* = 1.0 Hz, 1H), 8.02 (dd, *J* = 8.6,1.6 Hz, 1H), 7.79 (dd, *J* = 8.0,2.5 Hz, 1H), 7.55 (d, *J* = 8.6 Hz, 1H), 7.45 (dd, *J* = 8.9,4.2 Hz, 1H), 7.29-7.23 (m, 1H), 4.53 (t, *J* = 5.9 Hz, 2H), 3.89 (t, *J* = 5.9 Hz, 2H), 3.53-3.49 (m, 2H), 3.41-3.37 (m, 2H), 3.28 (s, 3H).

(E)-4-(2-(6-fluoro-9-(2-(2-methoxyethoxy)ethyl)-9H-carbazol-3-yl)vinyl)-1-(2-(2-methoxyethoxy)ethyl)pyridin-1-ium (F-SPG). A mixture of a solution of **3** (313 mg, 1 mmol), **8** (378 mg, 1 mmol) and piperidine (30 µL) in methanol (15 mL) was stirred at 40°C for 12 h. After solvent removal, **F-SPG** (347 mg) was obtained by precipitation from acetone in 56% yield. ^{1}H NMR (400 MHz, $MeOD_3$, δ): 8.68 (d, *J* = 7.0 Hz, 2H), 8.47 (s, 1H), 8.17-8.08 (m, 3H), 7.93-7.84 (m, 2H), 7.66 (d, *J* = 8.7 Hz, 1H), 7.60 (dd, *J* = 8.9,4.1 Hz, 1H), 7.42 (d, *J* = 16.2 Hz, 1H), 7.27 (td, *J* = 9.1,2.6 Hz, 1H), 4.65 (t, *J* = 4.7 Hz, 2H), 4.59 (t, *J* = 5.3 Hz, 2H), 3.96 (t, *J* = 4.9 Hz, 2H), 3.91 (t, *J* = 5.3 Hz, 2H), 3.66-3.62 (m, 2H), 3.52-3.48 (m, 4H), 3.39-3.35 (m, 2H), 3.31 (s, 3H), 3.19 (s, 3H). ^{13}C NMR (100 MHz, $CDCl_3$) δ 159.0, 156.7, 154.8, 143.9, 143.2, 137.7, 126.3, 126.2, 123.3, 123.2, 122.9, 122.8, 122.6, 121.7, 119.2, 113.7, 113.5, 110.7. 110.6, 110.2, 105.7, 105.4, 71.6, 71.5, 70.2, 69.9, 69.3, 68.9, 59.7, 57.8, 57.7, 43.2. HRMS (MALDI-TOF) *m/z* Calcd for $C_{29}H_{34}FN_2O_4$ 493.2497 Found 493.1337

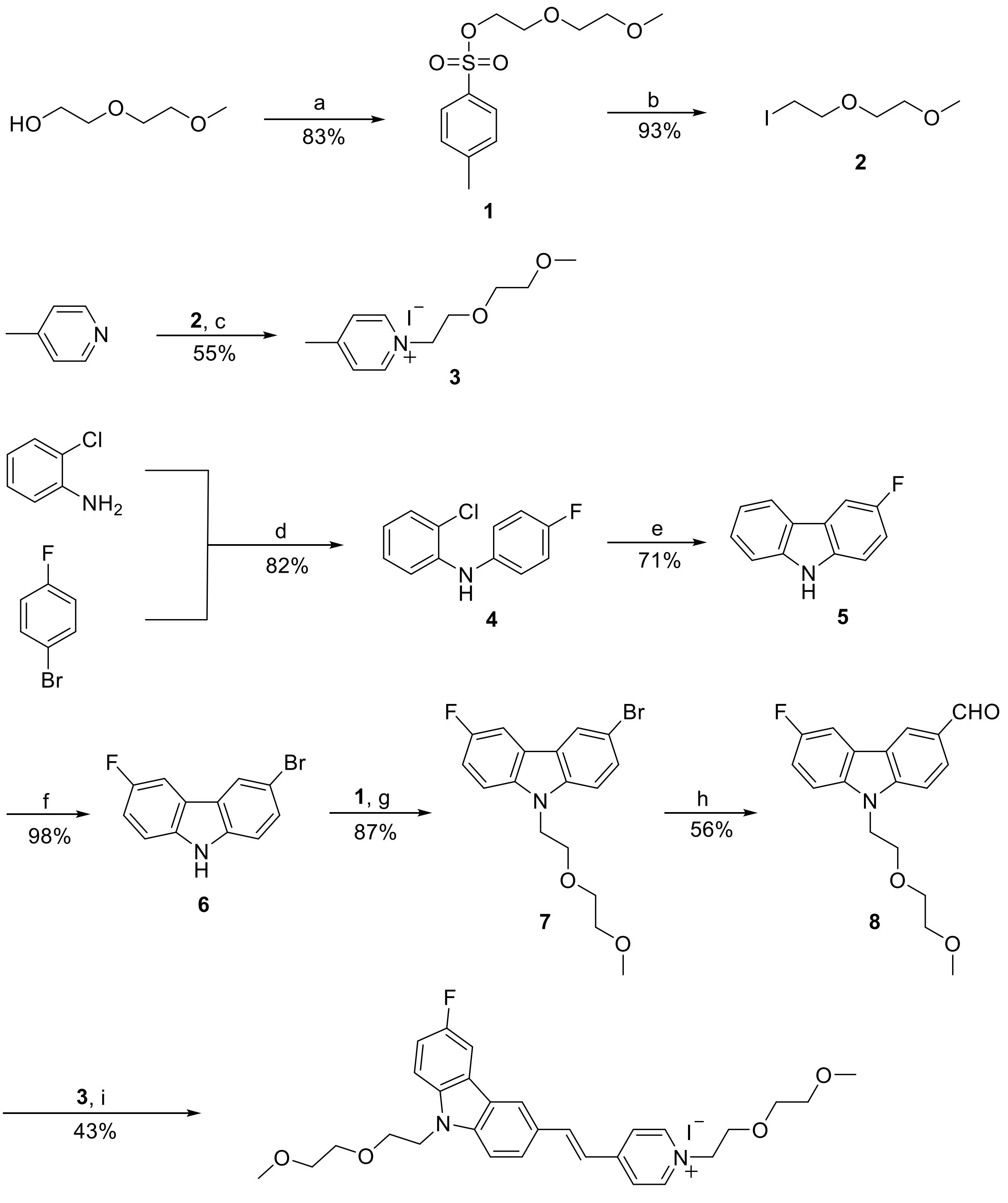

$[M]^+$.

F-SPG

Reagents and Condition: a) NaOH, tosyl chloride, THF/H_2O, 0 °C; b) NaI, Acetone, rt; c) AcN, 50 °C; d) NaOBu-*t*, $Pd(OAc)_2$, BINAP, toluene, reflux; e) K_2CO_3, $Pd(OAc)_2$, BINAP, DMF, 155 °C; f) NBS, THF, 0 °C; g) KOH, THF, reflux; h) (i) *n*-BuLi, THF (ii) N-formylmorpholine, -78 °C to r.t; i) piperidine, MeOH, 40 °C.

Scheme S1. The synthetic route of **F-SPG**.

^{1}H NMR Spectra of F-SPG

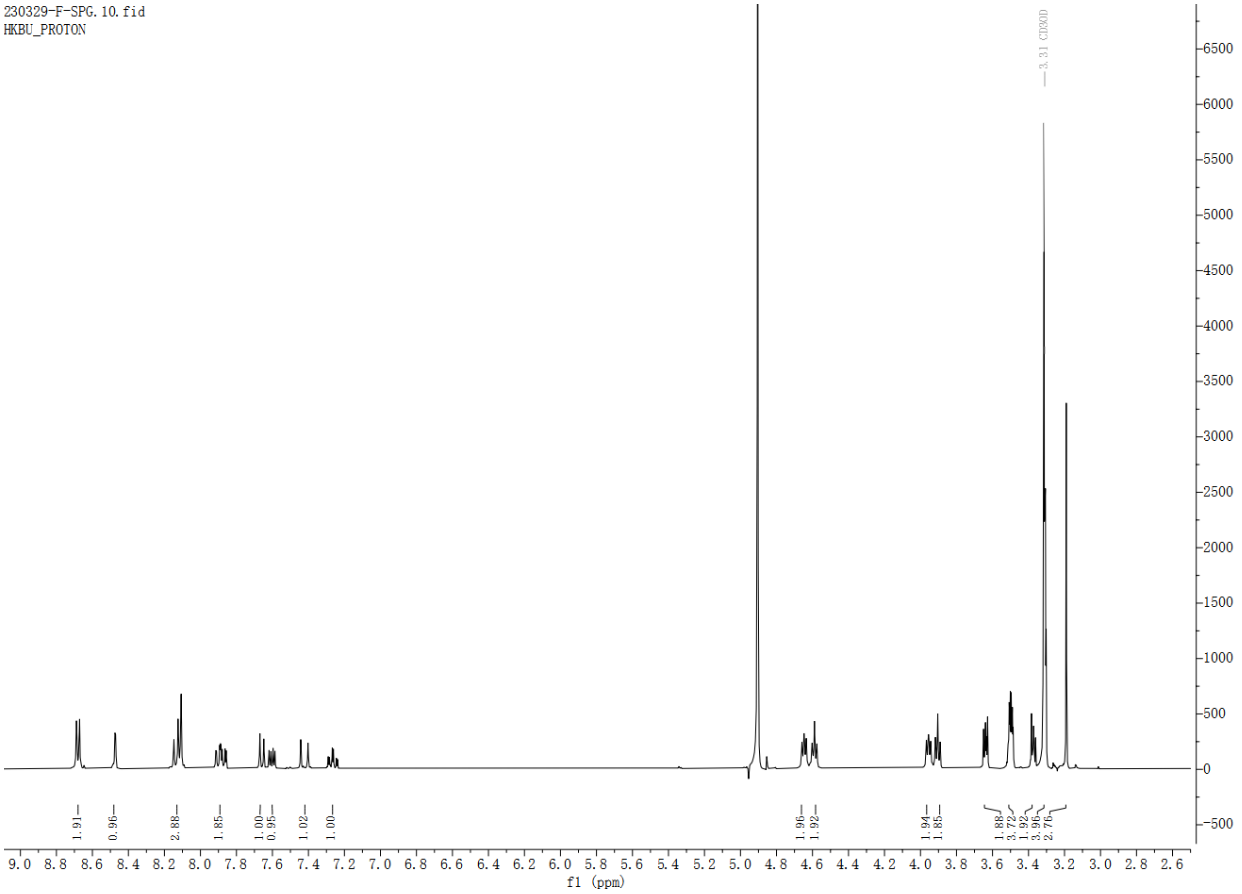

^{13}C NMR Spectra of F-SPG

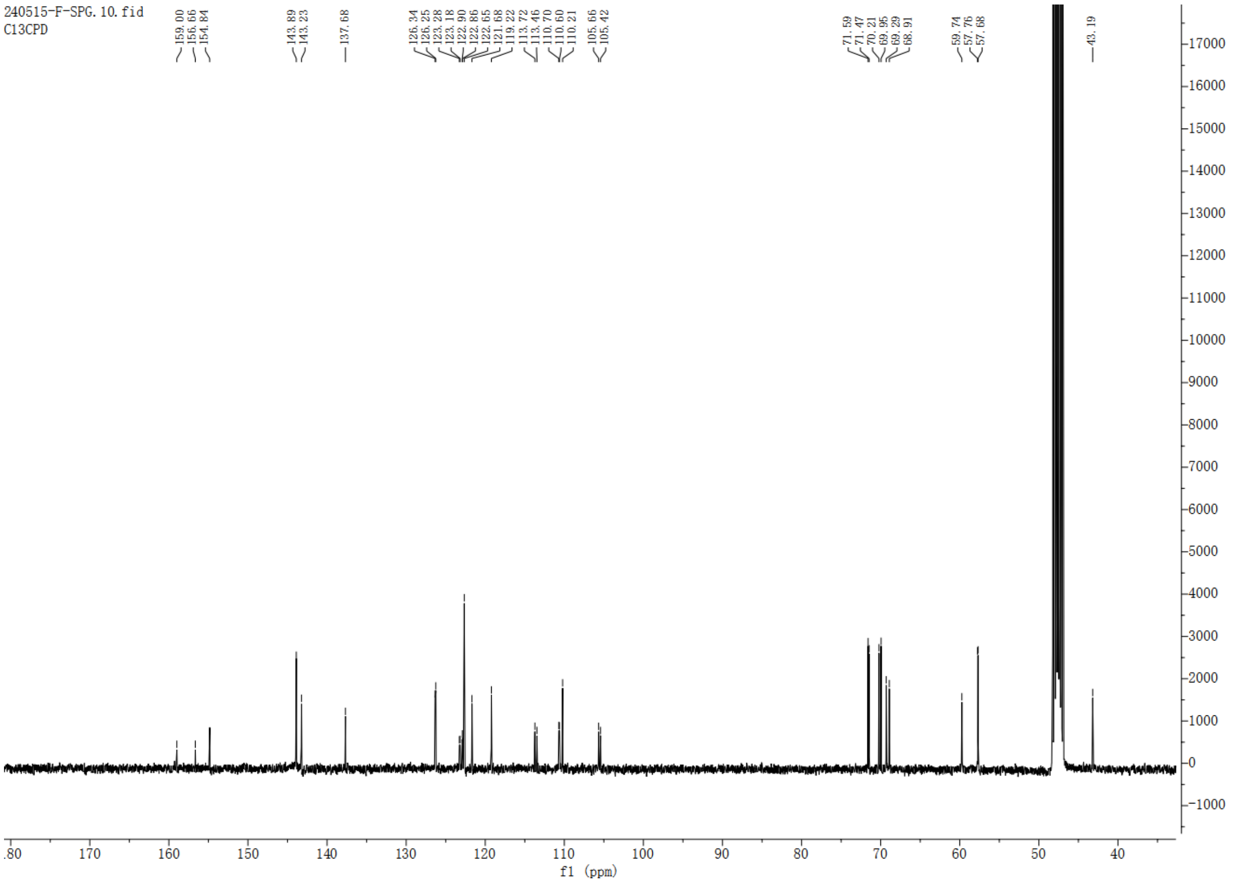

HRMS Spectra of F-SPG

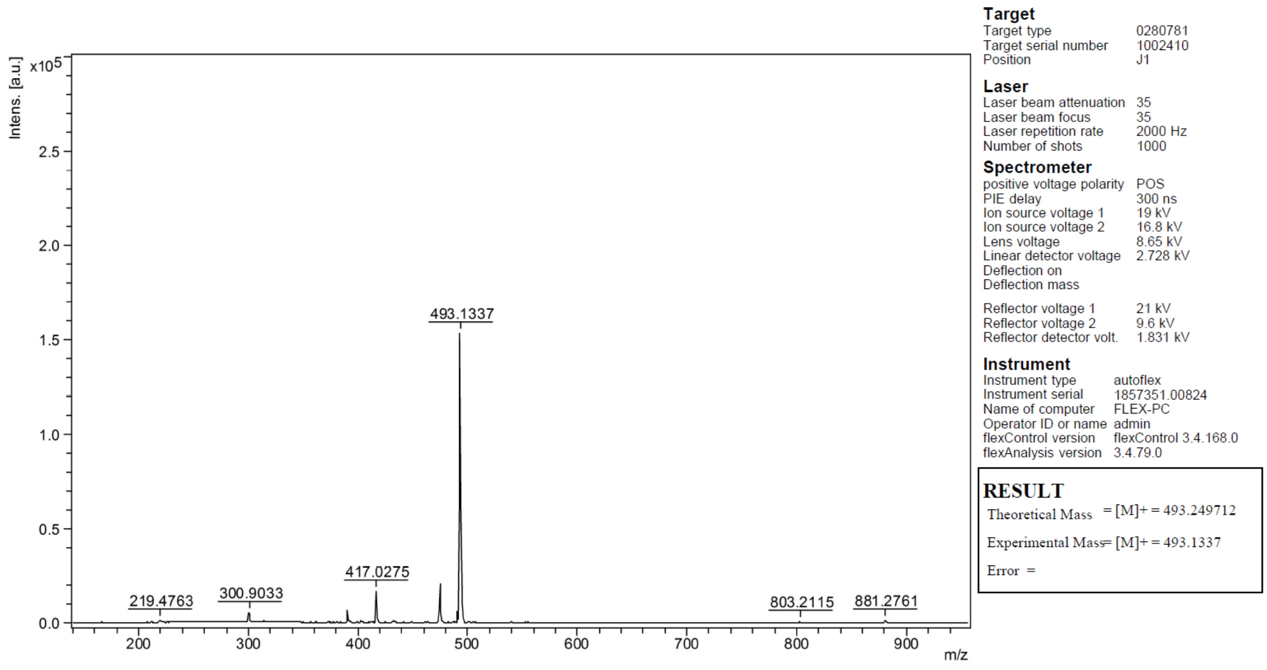

Figure S1. Characterization of **F-SPG**. ^{1}H NMR, ^{13}C NMR and HRMS Spectra of F-SPG.

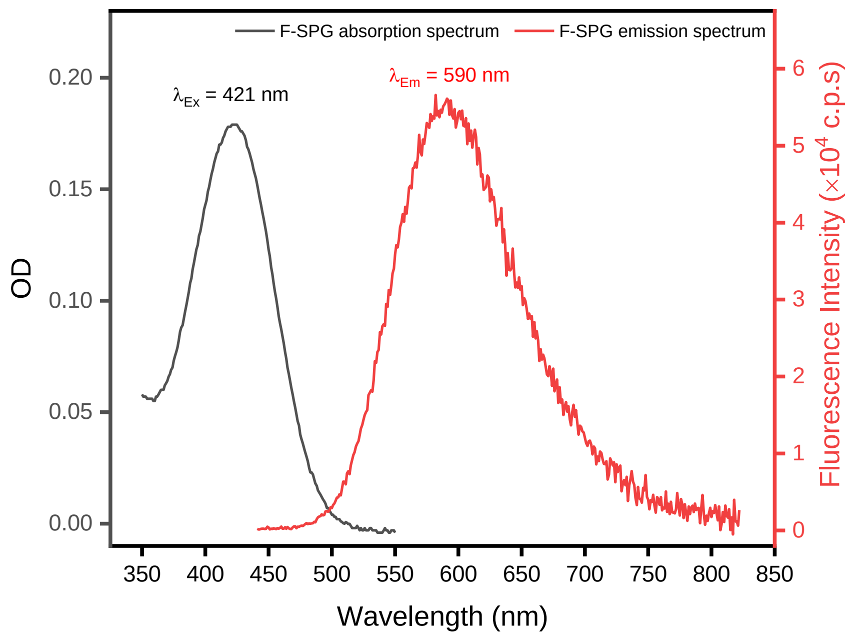

Figure S2. Absorption and emission spectrum of F-SPG in a pH 7.4 phosphate buffer.

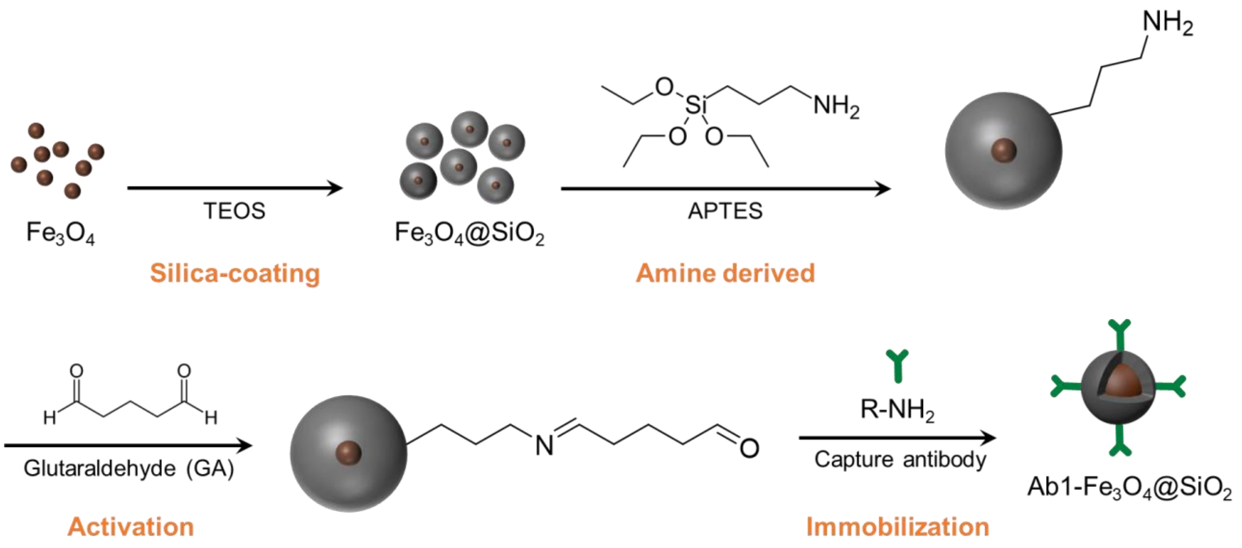

Figure S3. Preparation process of nanoprobes, Ab1-Fe_3O_4@SiO_2.

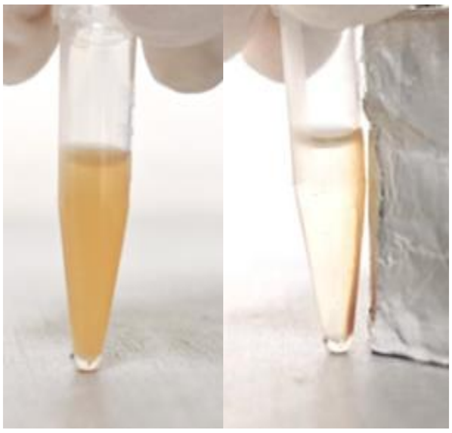

Figure S4. Magnetic properties of the nanoprobes.

References

1. Li, E.; Wang, K.; Zhang, B.; Guo, S.; Xiao, S.; Pan, Q., … Liu, B., Design, synthesis, and biological evaluation of novel carbazole derivatives as potent DNMT1 inhibitors with reasonable PK properties. *Journal of Enzyme Inhibition and Medicinal Chemistry*, **2022,** 37(1), 1537–1555.

2. Bedford, R.B.; Betham, M.; Charmant, J.P.H.; Weeks, A.L., Intramolecular direct arylation in the synthesis of fluorinated carbazoles, *Tetrahedron*, **2008**, 64(26), 6038-6050.

3. Iyaswamy A.; Wang, X.; Zhang, H.; Vasudevan, K.; Wankhar, D.; Lu, K.; Krishnamoorthi, S.; Guan, X.; Su, C.; Liu, J.; Kan, Y.; Jaganathan, R.; Deng, Z.; Li, H.; Wong M.S.; Li, M., Molecular engineering of a theranostic molecule that detects Aβ plaques, inhibits Iowa and Dutch mutation Aβ self-aggregation and promotes lysosomal biogenesis for Alzheimer's disease. *J. Mater. Chem. B*, **2024**,12, 7543-7556.
